# Comprehensive evaluation of the efficacy and patient acceptance of combined antidepressant and cognitive behavioral therapy in the treatment of post-traumatic stress disorder

**DOI:** 10.3389/fpsyt.2025.1737322

**Published:** 2026-01-12

**Authors:** Yanyan Jia, Zehua Xu, Jingming Yang, Jiabao Chai, Wei Li, Haiting Xu, Xiaohong Li

**Affiliations:** 1 Beijing Huilongguan Hospital, Peking University Huilongguan Clinical Medical College, Beijing, China; 2 Beijing Public Security Police Clinic, Beijing, China

**Keywords:** cognitive behavior therapy, drug therapy analysis, efficacy evaluation, patient acceptance, post-traumatic stress disorder treatment

## Abstract

**Background:**

To improve treatment outcomes and enhance the prognosis of patients with PTSD (Post-Traumatic Stress Disorder), the efficacy and patient acceptance of antidepressant treatment for PTSD are comprehensively evaluated, and its clinical application value is explored.

**Methods:**

A retrospective study is conducted, with 200 patients divided into a medication group and a combination group, with 100 patients in each group. The medication group receives a single antidepressant (primarily a selective serotonin reuptake inhibitor) for 8 weeks, while the combination group receives the same medication plus a 60-minute weekly trauma-focused cognitive behavioral therapy (TF-CBT). Symptom severity, side effects, and adherence are assessed using standardized clinical interviews and self-rating scales. Correlation analysis and multiple linear regression (MLR) are used to explore the relationships between these indicators.

**Results:**

The combination group has higher medication satisfaction (*p* < 0.001), lower frequency of side effects (insomnia: 0.70 ± 0.42 times *vs*. 1.25 ± 0.47 times, *p* < 0.001), and higher medication compliance rate (93.0% *vs*. 85.0%, *p* < 0.001).

**Conclusions:**

Compared with antidepressant monotherapy, combining antidepressant and CBT treatment significantly alleviates patient symptoms, reduces side effects, and improves treatment adherence and satisfaction. This study provides a new perspective for tailoring treatment plans and improving treatment precision and effectiveness.

## 1 Introduction

Post-traumatic stress disorder (PTSD) is a chronic mental disorder that occurs after experiencing extreme events (1, 2). It affects approximately 3.9% of adults globally, with the prevalence being even higher in certain high-risk populations (3). The treatment of PTSD primarily involves psychological interventions and pharmacotherapy. However, most current evaluation methods focus solely on the efficacy of drug treatment, often neglecting patients’ subjective experiences and treatment acceptability. This narrow focus makes it challenging to assess the overall effectiveness of treatment and lacks a robust scientific foundation.

Currently, mainstream international guidelines recommend trauma-focused psychotherapy and drug therapy as combined treatment options. In China, the prevention and treatment of PTSD also emphasizes the core role of psychotherapy. However, in clinical practice, single drug treatment is still relatively common, and patients’ subjective experience, satisfaction and long-term compliance with treatment are often ignored, resulting in poor treatment effects. Therefore, conducting a comprehensive evaluation of the efficacy and patient acceptance of PTSD drug treatment has important clinical significance. This study not only focuses on symptom relief, but also incorporates indicators such as patients’ subjective feelings, side effects, and compliance, aiming to comprehensively evaluate the real-world effects of the combined drug and psychological model. The results of the study can provide empirical evidence for clinicians to optimize treatment plans, improve patients’ treatment experience and long-term prognosis, and promote the development of PTSD treatment in China in a more humane and precise direction.

An integrated evaluation approach combining quantitative and qualitative methods is employed to assess the efficacy of PTSD treatment and patient acceptance. Evaluating outcomes from both clinical effectiveness and subjective experience perspectives provide clinicians with valuable insights into patient needs and support the refinement of treatment strategies, offering significant practical implications for future large-scale intervention research.

This study aims to retrospectively evaluate the efficacy and patient acceptance of antidepressant monotherapy versus antidepressant combined with cognitive behavioral therapy (CBT) in patients with post-traumatic stress disorder (PTSD). Specific objectives include: comparing the improvement of core PTSD symptoms (CAPS-5, PCL-5) between the two treatment models; assessing patient satisfaction with treatment, side effects, and medication compliance; exploring the association between efficacy indicators and patient acceptance, thereby providing empirical evidence for optimizing individualized treatment plans for PTSD.

## 2 Methods

### 2.1 Study participants and case screening

A total of 200 patients treated in a hospital between January and December 2023 are retrospectively selected as the study participants. Based on the diagnostic criteria for PTSD in the Diagnostic and Statistical Manual of Mental Disorders, Fifth Edition (DSM-5) (4–6).

#### 2.1.1 The case selection criteria are as follows

Exposure to a major traumatic event;

Meeting all DSM-5 diagnostic criteria for PTSD, including intrusive recollections, avoidance behaviors, altered negative emotions and cognitions, and heightened arousal, with symptoms persisting for more than one month and resulting in significant functional impairment;

Age range 18 to 65 years;

No psychotic disorder such as schizophrenia or bipolar disorder, and no severe personality disorder (such as borderline personality disorder);

Complete clinical records, including initial diagnosis and treatment records, and at least two follow-up visits.

#### 2.1.2 Situations that need to be excluded include

Current or recent (within the past six months) substance use disorder (such as alcohol or drug abuse);

Pregnancy or breastfeeding;

Known hypersensitivity to selective serotonin reuptake inhibitors or other drugs in treatment;

Inability to independently complete the assessment scale due to cognitive impairment or severe psychiatric symptoms;

Incomplete medical records refer to the lack of any of the following key data: assessment records at the initial diagnosis, medication records during treatment, scale scores, or post-test evaluation results at the end of treatment.

#### 2.1.3 Screening process

**Figure 1:**
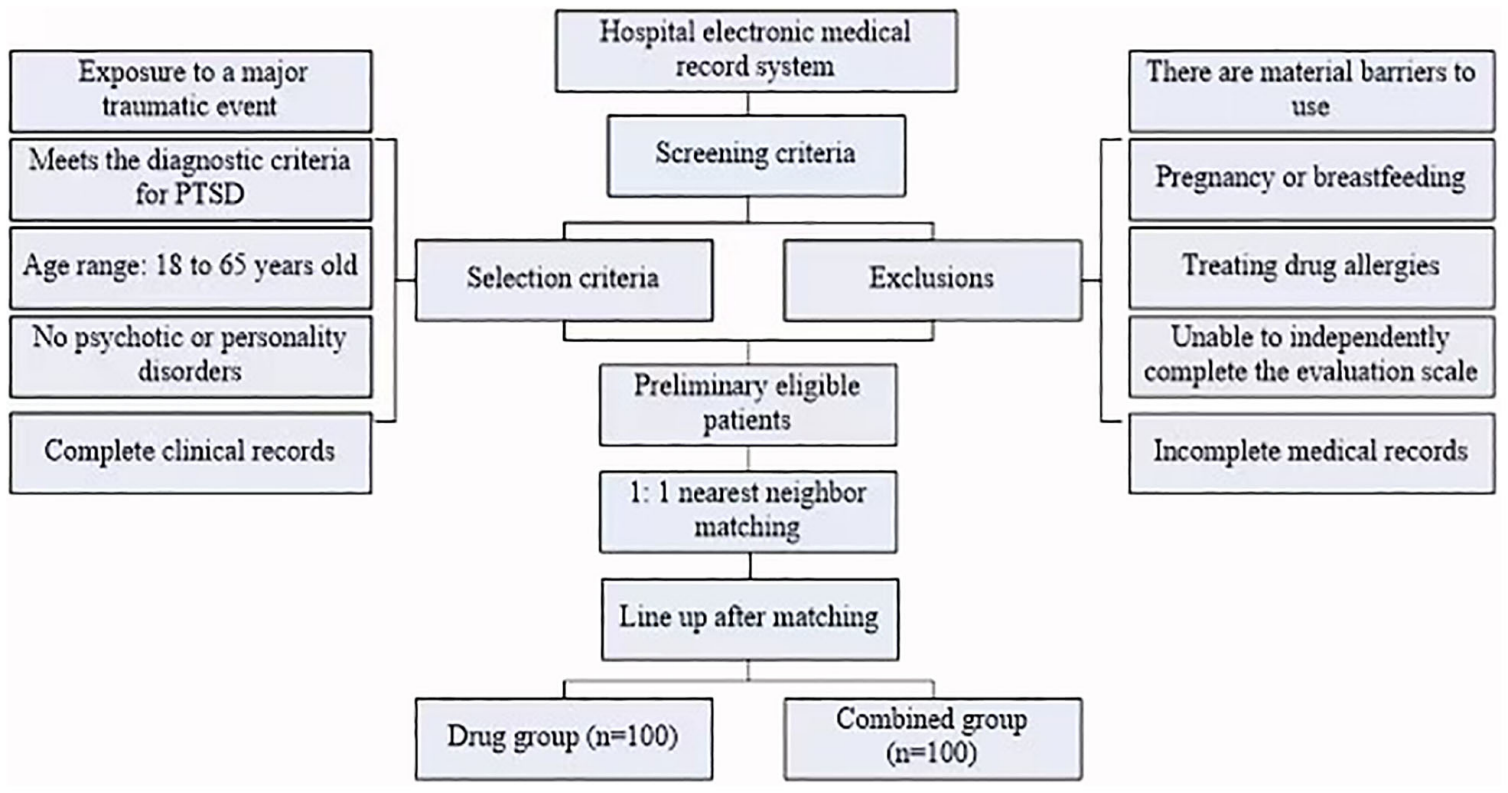
Patient selection process..

Two researchers independently review electronic medical records to identify potentially eligible patients based on the above criteria. For patients initially screened, the accuracy of diagnostic and treatment information is verified by verifying medical history records, psychological assessment reports, and laboratory test results. To control for potential confounding factors and enhance intergroup comparability, 1:1 nearest neighbor matching is used to pair the medication and combination groups. Matching variables include age, sex, trauma type, and baseline CAPS-5 score. The two researchers cross-validate the screening results, and any disagreements are resolved through discussion. Finally, from the eligible patients, 100 patients each in the medication and combination groups are selected for analysis based on the treatment regimen they received, as shown in **Figure 1**:

Before enrollment, all patients undergo a detailed clinical evaluation, including medical history collection, psychological testing, and laboratory tests. All 200 patients included in this study are outpatients in the psychiatric and psychological department of Beijing Huilongguan Hospital. All interventions are completed in the outpatient setting, and follow-up is conducted through appointments or telephone interviews. All patients sign an informed consent form to ensure transparency and voluntariness of participation in the evaluation (7).

### 2.2 Group design and treatment

200 PTSD patients are included in the study to evaluate the effect of drug therapy combined with psychological therapy on the efficacy of PTSD and patient acceptance. Through a retrospective study design, the 200 patients are divided into two groups according to the different treatment methods: the drug treatment group (drug group) and the drug combined with psychological intervention group (combined group), with 100 patients in each group. Both groups are assigned based on details in the medical records and cross-checked by two independent personnel. Patients in both groups were informed that they were receiving “standard PTSD treatment” without specifying the intervention type (e.g., the medication group was not told they were not receiving CBT), reducing expectation bias in self-reported outcomes (e.g., PCL-5, TSQM).

#### 2.2.1 Drug group

Patients in the drug group are treated with a single traditional antidepressive drug regimen (8–10), primarily a selective serotonin reuptake inhibitor(SSRIs), including sertraline (50-150mg/d), paroxetine (20-50mg/d), and fluoxetine (20-60mg/d). The specific medication was determined by physicians based on the patient’s age, liver and kidney function, and past medication history. All medications were administered once daily for 8 weeks. The medication regimen is formulated according to previous clinical guidelines. Each patient’s medication status is closely monitored, and dose adjustments, medication compliance, and adverse reactions are recorded.

#### 2.2.2 Combined group

Participants in the combined group receive standardized trauma-focused cognitive behavioral therapy (TF-CBT) (11–13) once weekly for 60 minutes for eight sessions, while taking antidepressants. Specific intervention components include:

Psychoeducation and Therapeutic Alliance Building: Participants are introduced to the etiology of PTSD and the principles of TF-CBT, and a personalized treatment plan is developed;

Cognitive Restructuring: Participants are identified as maladaptive beliefs related to trauma (such as self-blame and an overgeneralized sense of danger) and modified through Socratic questioning and evidence-based questioning;

Exposure and Processing of Traumatic Memories: Participants are guided to gradually confront traumatic memories through verbal retelling in a safe environment, reducing emotional avoidance;

Coping Skills Training: Participants are taught relaxation techniques, emotion regulation strategies, and problem-solving skills to enhance self-management skills.

All psychological interventions are delivered by full-time psychotherapists with a national Level II psychological counselor qualification. All therapists have received standardized, systematic training in trauma-focused TF-CBT (14, 15) and possess at least three years of clinical experience treating PTSD or related trauma-related disorders. Treatments are conducted using a standardized treatment manual, and senior supervisors regularly monitor quality to ensure consistent and standardized interventions.

### 2.3 Measurement tools

CAPS-5 (Clinician-Administered PTSD Scale for DSM-5): As the primary efficacy measure, it assesses the severity of core PTSD symptoms (16–18). This semi-structured clinical interview covers four dimensions: intrusive recollections, avoidance, negative emotions and cognitive changes, and heightened alertness. Scores are scored on a 0–4 scale (0 = no symptoms, 4 = very severe symptoms), with a total score range of 0-80, with higher scores indicating more severe symptoms.

PCL-5 (PTSD Checklist for DSM-5): As a secondary efficacy measure, it is self-assessed by patients (19–21). This 20-item scale corresponds to the four PTSD symptom clusters in DSM-5. Scores are scored on a 1–5 scale (1 = none, 5 = very severe), with a total score range of 20-100. It assesses the frequency of patients’ subjective symptoms.

TSQM (Treatment Satisfaction Questionnaire for Medication): This scale is specifically designed to measure patients’ subjective experience of medication treatment and encompasses four dimensions: effectiveness, side effects, convenience, and global satisfaction (22). Each group completes the first TSQM assessment after two weeks of treatment, followed by a second assessment in the final week of treatment. The scale uses a 1–7 point scale (1 being very dissatisfied and 7 being very satisfied). The survey is conducted anonymously to ensure the authenticity and reliability of patient feedback. To improve the effectiveness of the TSQM in clinical application, this study conducts brief interviews with patients in different groups after each questionnaire survey to better understand their acceptance of medication treatment.

### 2.4 Data collection and participant assessment process

Retrospective Study Design: This is a retrospective cohort study involving patients with PTSD who receive treatment at Beijing Huilongguan Hospital between January and December 2023 and complete an 8-week course of treatment. All patients are assigned to their treatment group based on their actual treatment regimen (pharmacology alone or medication combined with CBT), and data is collected from their complete electronic medical records. Although the intervention has concluded, pre-intervention (baseline) and post-intervention (week 8) assessment data is extracted according to a rigorous standardized process.

Assessment Timing and Assessors: All patients undergo a baseline assessment at their first visit by a dedicated assessment team, including history collection, DSM-5 diagnosis confirmation, and CAPS-5 and PCL-5 assessments. The baseline assessment is conducted by two clinical psychologists with at least five years of experience in PTSD assessment and an attending psychiatrist. All assessors receive standardized training in CAPS-5 assessment and are blinded to the patient’s group assignment during the study (i.e., assessors are not involved in the patient’s treatment decisions and are unaware of the patient’s group assignment during the post-test). The scale data before and after the intervention is fully recorded in the hospital electronic medical record system.

**Figure 2:**
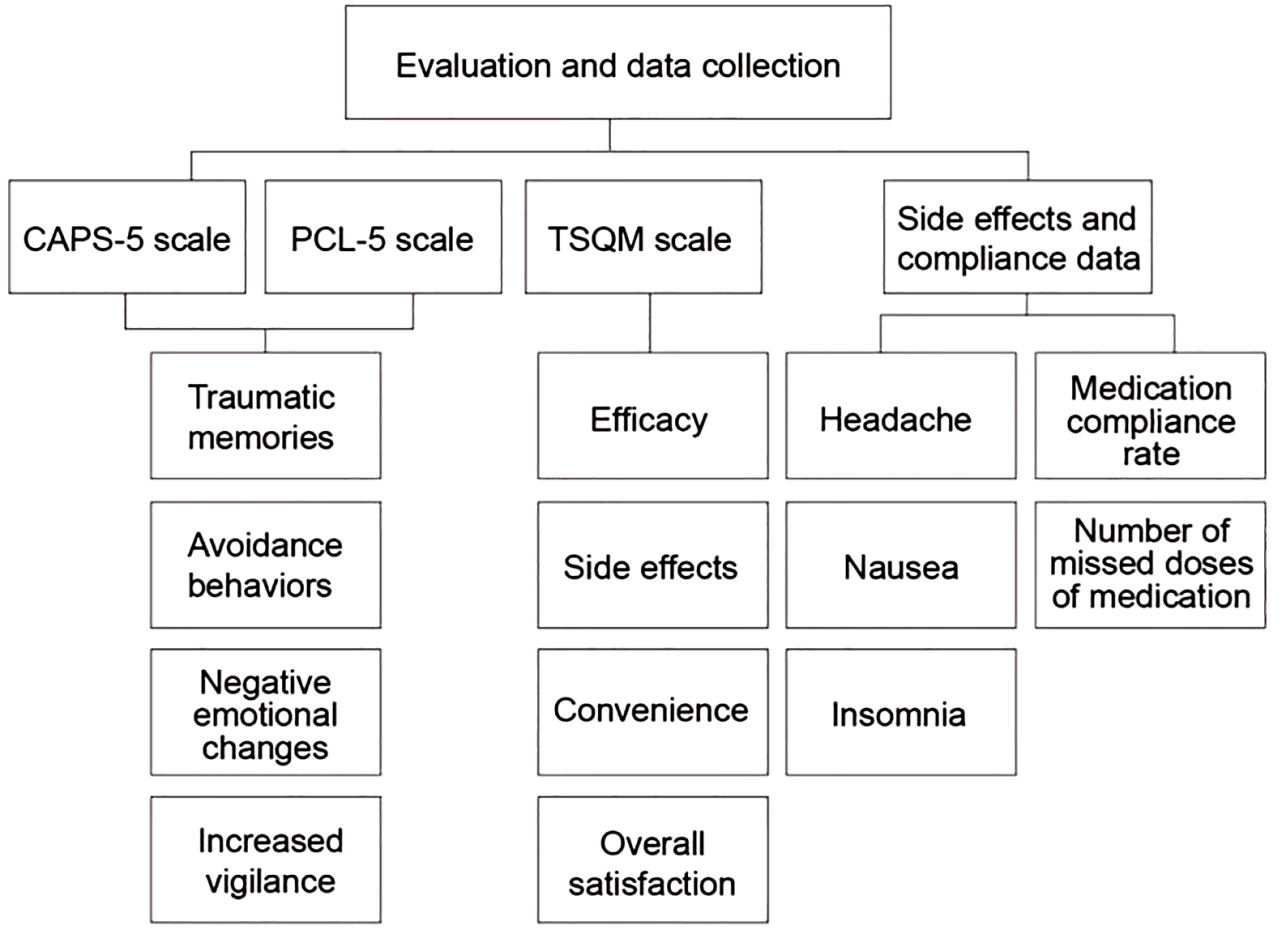
Evaluation and data collection.

**Figure 2** shows the evaluation and data collection of this paper:

Treatment Data Collection: TSQM Assessment: Patients complete the TSQM anonymously in a quiet, private space at Weeks 2 and 8 of treatment. After each assessment, a research assistant conducts a brief interview to collect qualitative feedback.

Side Effect Recording: Side effect data is collected using daily patient logs and regular clinic visits. The logs require patients to record the frequency of common adverse reactions, such as headache, nausea, and insomnia (23, 24).

Adherence Assessment: Medication diaries and interviews are used. Patients receive their medication packs and record their medication use at baseline. The logs are reviewed by research assistants at each follow-up visit, and in-depth interviews are conducted to understand reasons for missed doses and influencing factors.

Endpoint Assessment: At the end of Week 8, all patients are assessed with the CAPS-5 and PCL-5 post-tests by the same baseline assessment team (maintaining blinded status).

Data Management and Quality Control: All data are entered electronically using a double-entry method. Medical records are reviewed by two independent reviewers. The consistency of scale data is assessed using the Kappa test (CAPS-5, PCL-5) and Cronbach’s α coefficient (TSQM). All data are uploaded to an encrypted database to ensure security and integrity.

### 2.5 Data modeling and association analysis

Data modeling and association analysis aim to reveal the intrinsic relationship between drug efficacy and patient acceptance as a whole and provide quantitative support and a theoretical basis for rational clinical medication. This paper establishes an MLR model to conduct a preliminary analysis of indicators such as efficacy and acceptance and quantitatively evaluates the impact of each efficacy indicator on patient acceptance.

First, the collected efficacy data and acceptance data are cleaned and normalized. The efficacy data include patient CAPS-5 scores, PCL-5 scores, and other data, and the acceptance data includes the TSQM scale, treatment compliance, and other data. Multiple imputation method is used to fill in for missing data, and a complete data set is obtained based on the relationship between covariates. The data normalization process is:

(1) $$x^{\prime }=\frac{x-\mu }{\sigma }$$

In Equation 1, x is the raw data; μ is the mean; σ is the standard deviation. The standardized data is used for subsequent model analysis.

The Pearson correlation coefficient (PCC) method is used to measure the linear relationship between efficacy and patient acceptance:

(2) $$r=\frac{\sum_{i=1}^{n}\left(x_{i}-\bar{x}\right)\left(y_{i}-\bar{y}\right)}{\sqrt{\sum_{i=1}^{n}{\left(x_{i}-\bar{x}\right)}^{2}\sum_{i=1}^{n}{\left(y_{i}-\bar{y}\right)}^{2}}}$$

If |r|>0.5, it is considered a statistically significant correlation (Equation 2).

The MLR model is used to establish an efficacy prediction model further to explore the relationship between treatment effect and acceptance:

(3) $$y=\beta_{0}+\beta_{1}x_{1}+\beta_{2}x_{2}+\cdots +\beta_{n}x_{n}+\epsilon$$

In Equation 3, y represents the patient’s acceptance; ${\text{x}}_{\text{i}}$ represents the characteristic variable related to the treatment effect; ${\text{β}}_{\text{i}}$ represents the regression coefficient; ϵ represents the error. The regression coefficient is estimated using the ordinary least squares (Equation 4):

(4) $$\hat{\beta }={\left(X^{T}X\right)}^{-1}X^{T}y$$

### 2.6 Statistical analysis

SPSS 21.0 software is used for data analysis. The measurement data, such as age and CAPS-5 score, is expressed as mean and standard deviation, and the independent sample t test is used for inter-group comparison. The count data, such as gender and trauma type, is described as *n* (%) and compared using chi-square or non-parametric tests. Meanwhile, MLR analysis is used to further evaluate the comprehensive impact of multiple factors on patient acceptance. For all statistical tests, *p* < 0.05 indicates statistically significant differences. We used inverse probability of treatment weighting (IPTW) to adjust for unmeasured confounders, including trauma severity (assessed by additional clinical notes on trauma exposure intensity) and comorbidities (e.g., anxiety, depression comorbidity rates extracted from medical records).

## 3 Results

### 3.1 Descriptive analysis

To ensure the comparability of the two groups of patients at baseline, a comparative analysis of demographic and clinical characteristics is conducted, as shown in **Table 1**.

**Table 1:** Descriptive analysis of demographic and clinical characteristics (with standardized mean differences after IPTW).

Feature	Drug group (*n* = 100)	Combination group (*n* = 100)	Standardized Mean Difference (SMD)
Gender, *n* (%)			
Male	59 (59.0%)	56 (56.0%)	0.06
Female	41 (41.0%)	44 (44.0%)	0.06
**Age (years), mean ± SD**	38.4 ± 9.1	38.6 ± 8.7	0.02
Trauma type, *n* (%)			
Traffic accident	32 (32.0%)	30 (30.0%)	0.04
War or military conflict	2 (2.0%)	1 (1.0%)	0.08
Natural disaster	12 (12.0%)	15 (15.0%)	0.09
Serious bodily injury	33 (33.0%)	29 (29.0%)	0.08
Other	21 (21.0%)	25 (25.0%)	0.09
Initial CAPS-5 score (points), mean ± SD	65.4 ± 12.7	65.0 ± 12.3	0.03
Initial PCL-5 score (points), mean ± SD	52.3 ± 10.1	52.5 ± 9.9	0.02

All SMDs are< 0.1 after IPTW adjustment, confirming excellent balance between the drug group and combination group for all key demographic and clinical confounders. Bold values represent different classification criteria (gender, trauma type).

In **Table 1**, the two groups are similar in terms of gender distribution, mean age, trauma type, and pretreatment CAPS-5 and PCL-5 scores. Males account for 59.0% (59/100) of the medication group and 56.0% (56/100) of the combined treatment group. The mean ages of the two groups are 38.4 and 38.6 years, respectively, and the initial CAPS-5 scores are 65.4 and 65.0, respectively. These similarities in baseline characteristics indicate good comparability between the two groups and provide a basis for subsequent efficacy analyses.

### 3.2 Evaluation results of efficacy and acceptability

#### 3.2.1 CAPS-5 scale results

After different groups receive corresponding treatments, the efficacy and patient acceptance of each treatment are evaluated and analyzed. **Table 2** lists the changes in CAPS-5 scores before and after treatment.

**Table 2:** CAPS-5 scores before and after treatment.

Group	Number of cases (*n*)	Before treatment (points)	After treatment (points)
Drug group	100	65.4 ± 12.7	52.1 ± 10.2
Combined group	100	65.0 ± 12.3	39.5 ± 9.8
*t*		0.779	-12.312
*p*		0.853	<0.001

**Table 2** shows that the CAPS-5 scores of the two groups are similar before treatment, and there is no significant difference between the two groups (*p* = 0.853). After 8 weeks of treatment, the drug group scores 52.1 ± 10.2 points, and the combined group scores 39.5 ± 9.8 points. The comparison between the two groups shows that the CAPS-5 scores after treatment are significantly different (*p* < 0.001), indicating that the combined group has better efficacy and that the combined treatment of drugs and CBT can more effectively relieve PTSD symptoms and improve efficacy.

#### 3.2.2 PCL-5 scale results

**Table 3** shows the changes in PCL-5 scores before and after treatment.

**Table 3:** PCL-5 scores before and after treatment.

Group	Number of cases (*n*)	Before treatment (points)	After treatment (points)
Drug group	100	52.3 ± 10.1	37.8 ± 9.2
Combined group	100	52.5 ± 9.9	30.2 ± 8.7
*t*		0.152	-10.286
*p*		0.882	<0.001

From **Table 3**, the PCL-5 scores of the two groups are similar before treatment, and there is no statistically significant difference between the two groups (*p* = 0.882). After 8 weeks of treatment, the PCL-5 score of the drug group is 37.8 ± 9.2 points, and the PCL-5 score of the combined group is 30.2 ± 8.7 points. In the inter-group comparison, there are significant differences in the PCL-5 scores of the two groups after treatment, and the *p* value is<0.001, reaching the significance level. This result shows that combined therapy can improve the clinical symptoms of PTSD patients and the treatment effect.

#### 3.2.3 TSQM scale results

**Tables 4** and **5** show the TSQM scale results at weeks 2 and 8.

**Table 4:** TSQM scale scores at week 2.

Group	Number of cases (*n*)	Efficacy (points)	Side effects (points)	Convenience (points)	Overall satisfaction (points)
Drug group	100	4.35 ± 1.18	4.08 ± 0.80	4.26 ± 0.98	4.32 ± 1.25
Combined group	100	4.67 ± 1.01	4.60 ± 1.03	4.78 ± 0.77	4.78 ± 1.02
*t*		-4.323	-3.787	-3.570	-4.091
*p*		<0.001	<0.001	<0.001	<0.001

**Table 5:** TSQM scale scores at week 8.

Group	Number of cases (*n*)	Efficacy (points)	Side effects (points)	Convenience (points)	Overall satisfaction (points)
Drug group	100	4.52 ± 1.15	4.35 ± 0.88	4.58 ± 0.96	4.58 ± 1.20
Combined group	100	5.09 ± 1.01	4.93 ± 1.05	5.12 ± 0.72	5.25 ± 0.98
*t*		-6.725	-4.447	-3.292	-4.744
*p*		<0.001	<0.001	<0.001	<0.001

From the results in **Table 4**, at week 2, the scores of the drug group in the four items of efficacy, side effects, convenience, and overall satisfaction are 4.35 ± 1.18 points, 4.08 ± 0.80 points, 4.26 ± 0.98 points, and 4.32 ± 1.25 points, respectively. In comparison, the scores of the combined group in the four items are 4.67 ± 1.01 points, 4.60 ± 1.03 points, 4.78 ± 0.77 points, and 4.78 ± 1.02 points. In the inter-group comparison, there are significant differences in the scores of treatment effect, side effects, convenience, and overall satisfaction between the groups after receiving treatment. The *p* values of each item are all less than 0.001, reaching the significance level, indicating that the combined group has an ideal overall treatment experience for patients.

From the results in **Table 5**, at week 8, the scores of the drug group in the four items are 4.52 ± 1.15, 4.35 ± 0.88, 4.58 ± 0.96, and 4.58 ± 1.20, respectively, while the scores of the combined group in the four items are 5.09 ± 1.01, 4.93 ± 1.05, 5.12 ± 0.72, and 5.25 ± 0.98, respectively. The combined group scores significantly higher than the medication group on all dimensions of the TSQM. This result suggests that patients receiving combined CBT treatment report greater satisfaction and acceptance of their antidepressant medication. This may be related to CBT indirectly enhancing patients’ positive experience with medication by improving overall symptoms, increasing confidence in treatment, or improving self-management skills. It is important to emphasize that the TSQM assesses patient satisfaction with medication, not acceptance of psychotherapy itself.

#### 3.2.4 Side effects and compliance results

The side effects and compliance results are shown in **Tables 6** and **7**.

**Table 6:** Side effect results.

Group	Number of cases (*n*)	Headache	Nausea	Insomnia
Drug group	100	1.21 ± 0.45	0.76 ± 0.32	1.25 ± 0.47
Combined group	100	0.90 ± 0.40	0.55 ± 0.35	0.70 ± 0.42
*t*		3.247	3.754	6.051
*p*		<0.001	<0.001	<0.001

**Table 7:** Compliance results.

Group	Number of cases (*n*)	Medication compliance rate (%)	Number of missed doses of medication
Drug group	100	85.0 ± 10.5	1.20 ± 1.50
Combined group	100	93.0 ± 10.0	0.80 ± 1.20
*t*		-3.669	5.271
*p*		<0.001	<0.001

From **Table 6**, the frequencies of side effects of headache, nausea, and insomnia in the drug group are 1.21 ± 0.45 times, 0.76 ± 0.32 times, and 1.25 ± 0.47 times, respectively. In contrast, the frequencies of side effects of headache, nausea, and insomnia in the combined group are 0.90 ± 0.40 times, 0.55 ± 0.35 times, and 0.70 ± 0.42 times, respectively. In the inter-group comparison, there are significant differences in the frequencies of side effects between the groups after receiving treatment, and their *p* values are all less than 0.001.

From **Table 7**, the medication compliance rate of the drug group is 85.0 ± 10.5%, and the number of missed medications is 1.20 ± 1.50 times. In contrast, the medication compliance rate of the combined group is 93.0 ± 10.0%, and the number of missed medications is 0.80 ± 1.20 times. The *p* values of the inter-group comparison are all less than 0.001, and the compliance results are significantly different. This result shows that the medication compliance rate and the number of missed medications of the combined group patients are significantly improved. CBT can effectively improve patients’ treatment compliance and efficacy by improving their treatment motivation and self-management ability.

### 3.3 Correlation analysis

Based on the results of efficacy and acceptability evaluation, a correlation analysis is conducted, and **Table 8** shows the results.

**Table 8:** Correlation analysis results.

Variable	CAPS-5 score	PCL-5 score	TSQM score	Side effect results	Compliance results
CAPS-5 score	1.000	0.95*	0.87*	-0.56*	0.73*
PCL-5 score	0.95*	1.000	0.88*	-0.51*	0.70*
TSQM score	0.87*	0.88*	1.000	-0.47*	0.79*
Side effect results	-0.56*	-0.51*	-0.47*	1.000	-0.62*
Compliance results	0.73*	0.70*	0.79*	-0.62*	1.000

TSQM score refers to the total scale score at Week 8 of treatment.* indicates statistical significance.

In **Table 8**, the scores of CAPS-5 and PCL-5 show a high positive correlation (*r* = 0.95, *p* < 0.05). This result indicates that the scores of CAPS-5 and PCL-5 change in the same direction, suggesting that the efficacy of drug therapy combined with psychological therapy for PTSD is consistent in clinical evaluation and patient self-evaluation. The analysis results showed that the TSQM score at Week 8 was significantly positively correlated with CAPS-5 score and PCL-5 score (*r* = 0.87, *r* = 0.88, *p* < 0.05), which means that as PTSD symptoms are alleviated, patient acceptance also increases, further confirming a close relationship between the efficacy of combination therapy and patient acceptance. CAPS-5, PCL-5, and the frequency of adverse reactions are negatively correlated (*r* = -0.56, *r* = -0.51, *p* < 0.05), indicating that the more effective the treatment method is, the fewer adverse reactions the patient may suffer. Patient compliance correlates significantly positively with CAPS-5 and PCL-5 scores (*r* = 0.73, *r* = 0.70, *p* < 0.05). The higher the patient’s compliance during medication, the better the efficacy, indicating that compliance positively impacts efficacy. The incidence of side effects negatively correlates with compliance results (*r* = -0.62, *p* < 0.05), indicating that the more adverse reactions occur, the worse the compliance. The increase in adverse drug reactions can cause patients to interrupt or fail to take medication in time during medication, thus affecting the efficacy.

### 3.4 MLR

This paper takes the patient’s treatment acceptance as the dependent variable and the efficacy index as the independent variable to further explore the mechanism between efficacy and acceptance. The four dimensions of the TSQM scale quantify the patient acceptance. The efficacy index is evaluated by CAPS-5 and PCL-5 scales. **Table 9** shows the final regression analysis results:

**Table 9:** MLR analysis results (with VIF values).

Independent variable	Regression coefficient *β*	Standard error (SE)	*t*	*p*	Variance Inflation Factor (VIF)
CAPS-5 score	0.45	0.126	3.752	0.001	3.45
PCL-5 score	0.32	0.103	3.204	0.001	3.28
Efficacy	0.32	0.085	4.226	0.027	2.15
Side effects	-0.18	0.131	-2.227	<0.001	1.82
Convenience	0.38	0.096	4.594	0.019	2.07
Overall satisfaction	0.41	0.111	3.736	0.001	2.94

All VIF values range from 1.82 to 3.45 (all< 5), indicating no moderate or severe multicollinearity in the model.

In **Table 9**, the effects of the CAPS-5 score, PCL-5 score, efficacy, side effects, convenience, and overall satisfaction on treatment acceptance are statistically significant, and their *p* values are all less than 0.05. Among them, the regression coefficient of side effects is negative (*β* = -0.18, *p* < 0.001), indicating that the increase in side effects and the decrease in efficacy are statistically significant, which means that the frequency of side effects has an essential impact on the patient’s efficacy. In addition to the efficacy, the patient’s acceptance of treatment is also affected by side effects, convenience, and overall satisfaction.

## 4 Discussion

Existing studies have preliminarily explored the potential of combining antidepressants with psychotherapy in the management of PTSD (25–27). Merz et al. compared the efficacy and acceptability of psychotherapy, drug therapy, and their combination in adult PTSD patients through a network meta-analysis. The results showed that antidepressants combined with psychotherapy were superior to single treatment models in symptom relief, but the study lacked long-term follow-up data and did not conduct an in-depth analysis of patients’ subjective acceptance of treatment (28). This finding suggests that combined treatment may have advantages, but its tolerability, satisfaction, and compliance in the real world still need further verification. To further identify the Intermediate Phenotype (IP) of PTSD and assess the sensitivity of IP to the treatment of post-traumatic stress symptoms, Palmisano et al. searched five databases for empirical studies published in English between January 2010 and August 1, 2022. They provided preliminary support for the utility of IP measures in assessing treatment efficacy, but the risk of bias and methodological limitations limited the validity and generalizability of the results (29). Cusack et al. studied the effect of General Self-Efficacy (GSE) on the efficacy of PTSD and used multilevel modeling to examine archival data of male veterans receiving mental health services. The results showed that efforts to improve GSE and focus on moderating factors such as educational attainment may help improve the treatment of PTSD (30). Existing research has made important progress in the evaluation of the efficacy of PTSD treatment and patient acceptance, providing rich data and methodological support. However, it ignores individual differences and pays less attention to patients’ subjective experience and treatment compliance. There are problems of methodological bias and insufficient generalizability of the results.

Patient acceptance is an essential factor in the successful treatment of PTSD, which directly affects treatment compliance and long-term efficacy (31–33). Zhao et al. investigated the relationship between self-acceptance, PTSD, and post-traumatic growth (PTG) among Chinese rescuers by examining the mediating role of social support. The results showed that the model adequately fit the data and indicated that social support partially mediated the relationship between self-acceptance, PTSD, and PTG (34). Roche used a case study to illustrate the management of PTSD using an Acceptance and Commitment Therapy (ACT)-based approach in the context of Traumatic Brain Injury (TBI). Outcome measures were taken before and after the intervention and at 3-month and 12-month follow-up. ACT outcome indicators, psychological indicators, and quality of life scores all improved, consistent with subjective reports, indicating that acceptance and commitment therapy is a feasible intervention for treating post-traumatic stress disorder after TBI (35). Kelly et al. conducted a pilot randomized trial of the effect of an ACT-based approach in improving social support for veterans with PTSD. Baseline assessment and follow-up results showed that participants in the ACT group had significant improvements in the quality of social relationships, participation in social and leisure activities, and symptoms of post-traumatic stress disorder (36). Existing studies have different focuses on the evaluation of efficacy and patient acceptance in PTSD treatment, but lack systematicity and comprehensiveness.

Comprehensive assessments in this study show that compared with the medication group, the combined group performs better in all dimensions of the CAPS-5, PCL-5, and TSQM (Satisfaction with Antidepressants), with fewer side effects and higher compliance. This suggests that combining antidepressants with CBT not only more effectively alleviates core PTSD symptoms but also significantly improves patient satisfaction with and adherence to antidepressants. To minimize differential care, we emphasized that all therapists followed a standardized TF-CBT manual, and senior supervisors conducted monthly audits to ensure consistent intervention delivery (audit results showed >95% adherence to the manual). This increased medication acceptance may be due to CBT improving patients’ overall psychological state and treatment motivation through mechanisms such as cognitive restructuring and coping skills training, thereby indirectly enhancing their positive evaluation of medication. Cognitive restructuring techniques in CBT may help patients view medication effects more rationally, reduce disappointment caused by inflated expectations or misunderstandings, and thus enhance their perception of medication efficacy (TSQM-Effectiveness dimension). The coping skills and emotion regulation strategies taught in CBT may buffer common initial medication side effects (such as activation and insomnia), making patients more tolerable to adverse reactions (TSQM-Side Effects dimension), thereby improving adherence. Furthermore, regular psychological counseling itself may enhance patients’ motivation for treatment and sense of social support. This positive therapeutic alliance may generalize to trust and acceptance of the entire treatment plan (including medication). Therefore, CBT not only directly targets core PTSD symptoms but may also indirectly amplify the net benefits of medication treatment by improving patients’ psychosocial functioning and treatment engagement.

Based on the evaluation and analysis results, it is recommended that health education and psychological counseling for patients in clinical work be strengthened to improve patient compliance and satisfaction. In the early stages, patients should be given detailed medication guidance, and regular follow-up and psychological counseling can be used to improve their self-care ability. At the same time, the side effects produced during their treatment should be actively evaluated to reduce their adverse reactions and improve the overall treatment experience. During treatment, physicians should pay attention to patients’ feedback and optimize it to enhance patient satisfaction and achieve better efficacy. Future research can further explore the acceptance of treatment in different populations, evaluate the effectiveness of individualized treatment, and provide new ideas and approaches for the diversified treatment of PTSD.

Although this study provides evidence for the effectiveness of combining medication with CBT for the treatment of PTSD, several limitations remain. This is a single-center, retrospective cohort study with a relatively limited sample size and a lack of long-term follow-up data, preventing the assessment of the long-term effects of combined treatment. We acknowledge the risk of single center bias in this study, as all participants were outpatients from a hospital in Beijing. The results may not be applicable to rural populations or hospitalized patients with severe comorbidities. We suggest conducting multicenter studies involving different clinical environments in the future. The sample size is insufficient for subgroup analysis (e.g. by trauma type or age), and plan to explore these subgroups in future multicenter studies using larger samples (≥ 500 participants). We acknowledge that post-traumatic stress disorder is a chronic disease, and 8 weeks is not sufficient to assess long-term recurrence rates and sustained compliance. We plan to conduct a 12-month follow-up of the research cohort and report long-term results in subsequent manuscripts. This study did not use specialized assessment tools for psychological interventions, and only indirectly reflected patients’ acceptance of CBT through the TSQM scale (focusing on medication satisfaction) and treatment adherence. This may not fully capture patients’ subjective experiences of psychotherapy itself. Future studies can introduce tools such as psychotherapy satisfaction scales and therapeutic alliance scales to more directly and accurately assess patients’ acceptance of CBT and related influencing factors. There is a risk of indication confusion in this study, acknowledging that clinical treatment decisions may be influenced by unrecorded patient factors such as family support and treatment willingness. Finally, the sample is primarily drawn from outpatients, and the results may not be applicable to inpatients with more severe or complex comorbidities.

## 5 Conclusions

This study explores the efficacy of pharmacotherapy for PTSD and patient acceptance of treatment using scale assessment, correlation analysis, and MLR analysis. Results show that combining antidepressants with CBT significantly alleviates symptoms, reduces side effects, and improves patient satisfaction and adherence with antidepressants compared to pharmacotherapy alone. This suggests that psychological interventions may indirectly enhance patient acceptance of pharmacotherapy by improving the overall treatment experience. This paper provides new ideas for the comprehensive treatment and evaluation of PTSD, emphasizing the comprehensive role of treatment effect and the patient’s overall experience.

## Data Availability

The raw data supporting the conclusions of this article will be made available by the authors, without undue reservation.

## References

[1] Barron I Freitas F Bosch CA . Pilot randomized control trial: efficacy of a group-based psychosocial program for youth with PTSD in the Brazilian favelas. J Child Adolesc Trauma. (2020) 14:335–45. doi: 10.1007/s40653-020-00328-8, PMID: 34471452 PMC8357894

[2] Belleville G Ouellet M-C Békés V Lebel J Morin CM Bouchard S . Efficacy of a therapist-assisted self-help internet-based intervention targeting PTSD, depression, and insomnia symptoms after a disaster: A randomized controlled trial. Behav Ther. (2023) 54:230–46. doi: 10.1016/j.beth.2022.08.004, PMID: 36858756

[3] Hood CO Southward MW Bugher C Sauer-Zavala S . A Preliminary Evaluation of the Unified Protocol among Trauma-Exposed Adults with and without PTSD. Int J Environ Res Public Health. (2021) 18:11729–40. doi: 10.3390/ijerph182111729, PMID: 34770243 PMC8583442

[4] Rumball F Happé F Grey N . Experience of trauma and PTSD symptoms in autistic adults: risk of PTSD development following DSM-5 and non-DSM-5 traumatic life events. Autism Res. (2020) 13:2122–32. doi: 10.1002/aur.2306, PMID: 32319731

[5] Forkus SR Raudales AM Rafiuddin HS Weiss NH Messman BA Contractor AA . The posttraumatic stress disorder (PTSD) checklist for DSM-5: A systematic review of existing psychometric evidence. Clin Psychol. (2023) 30:110–21. doi: 10.1037/cps0000111, PMID: 37378352 PMC10292741

[6] Zuromski KL Ustun B Hwang I Keane TM Marx BP Stein MB . Developing an optimal short-form of the PTSD Checklist for DSM-5 (PCL-5). Depression Anxiety. (2019) 36:790–800. doi: 10.1002/da.22942, PMID: 31356709 PMC6736721

[7] Geier TJ Hunt JC Nelson LD Brasel KJ deRoon-Cassini TA . Detecting PTSD in a traumatically injured population: The diagnostic utility of the PTSD Checklist for DSM-5. Depression Anxiety. (2019) 36:170–8. doi: 10.1002/da.22873, PMID: 30597679 PMC6373876

[8] Schrader C Ross A . A review of PTSD and current treatment strategies. Missouri Med. (2021) 118:546–51., PMID: 34924624 PMC8672952

[9] Thakur A Choudhary D Kumar B Chaudhary A . A review on post-traumatic stress disorder (PTSD): symptoms, therapies and recent case studies. Curr Mol Pharmacol. (2022) 15:502–16. doi: 10.2174/1874467214666210525160944, PMID: 34036925

[10] Akiki TJ Abdallah CG . Are there effective psychopharmacologic treatments for PTSD? J Clin Psychiatry. (2018) 80:1309–10. doi: 10.4088/JCP.18ac12473, PMID: 30695292 PMC6436624

[11] Bourdon D-É El-Baalbaki G Girard D Lapointe-Blackburn É Guay S . Schemas and coping strategies in cognitive-behavioral therapy for PTSD: A systematic review. Eur J Trauma Dissociation. (2019) 3:33–47. doi: 10.1016/j.ejtd.2018.09.005

[12] Mohajerin B Lynn SJ Cassiello-Robbins C . Unified protocol vs trauma-focused cognitive behavioral therapy among adolescents with PTSD. Behav Ther. (2023) 54:823–38. doi: 10.1016/j.beth.2023.03.003, PMID: 37597960 PMC10060014

[13] Ascienzo S Sprang G Royse D . Gender differences in the PTSD symptoms of polytraumatized youth during isolated phases of trauma-focused cognitive behavioral therapy. psychol trauma: theory research Pract Policy. (2022) 14:488–96. doi: 10.1037/tra0001028, PMID: 33617283

[14] Held P Bagley JM Klassen BJ Pollack MH . Intensively delivered cognitive-behavioral therapies: an overview of a promising treatment delivery format for PTSD and other mental health disorders. Psychiatr Ann. (2019) 49:339–42. doi: 10.3928/00485713-20190711-01

[15] McGeary DD Resick PA Penzien DB McGeary CA Houle TT Eapen BC . Cognitive behavioral therapy for veterans with comorbid posttraumatic headache and posttraumatic stress disorder symptoms: A randomized clinical trial. JAMA Neurol. (2022) 79:746–57. doi: 10.1001/jamaneurol.2022.1567, PMID: 35759281 PMC9237802

[16] Lee DJ Weathers FW Thompson-Hollands J Sloan DM Marx BP . Concordance in PTSD symptom change between DSM-5 versions of the Clinician-Administered PTSD Scale (CAPS-5) and PTSD Checklist (PCL-5). psychol Assess. (2022) 34:604–9. doi: 10.1037/pas0001130, PMID: 35389681 PMC9437843

[17] Oliveira-Watanabe TT Ramos-Lima LF Santos RC Mello MF Mello AF . The clinician-administered PTSD scale (CAPS-5): adaptation to Brazilian portuguese. Rev Bras psiquiatria (Sao Paulo Brazil: 1999). (2019) 41:92–3. doi: 10.1590/1516-4446-2018-0136, PMID: 30758463 PMC6781712

[18] Kim J-M Kim J-W Kang H-J Lee J-Y Jang H Jeong I . Assessing the predictive validity of early post-injury CAPS-5 for later posttraumatic stress disorder diagnosis. J Clin Psychiatry. (2024) 85:56272–9. doi: 10.4088/JCP.24m15267, PMID: 39145677

[19] Kramer LB Whiteman SE Petri JM Spitzer EG Weathers FW . Self-rated versus clinician-rated assessment of posttraumatic stress disorder: an evaluation of discrepancies between the PTSD checklist for DSM-5 and the clinician-administered PTSD scale for DSM-5. Assessment. (2023) 30:1590–605. doi: 10.1177/10731911221113571, PMID: 35915927

[20] Stanley IH Tock JL Boffa JW Hom MA Joiner TE . Psychometric properties of the PTSD Checklist for DSM-5 (PCL-5) anchored to one’s own suicide attempt. psychol trauma: theory research Pract Policy. (2024) 16:425–34. doi: 10.1037/tra0001456, PMID: 36862477

[21] Lee DJ Bovin MJ Weathers FW Palmieri PA Schnurr PP Sloan DM . Latent factor structure of DSM-5 posttraumatic stress disorder: Evaluation of method variance and construct validity of novel symptom clusters. psychol Assess. (2019) 31:46–58. doi: 10.1037/pas0000642, PMID: 30113182 PMC6312504

[22] Atkinson MJ Kumar R Cappelleri JC Hass SL . Hierarchical construct validity of the treatment satisfaction questionnaire for medication (TSQM version II) among outpatient pharmacy consumers. Value Health. (2005) 8 Suppl 1:S9–S24. doi: 10.1111/j.1524-4733.2005.00066.x, PMID: 16336491

[23] Hoppen TH Lindemann AS Morina N . Safety of psychological interventions for adult post-traumatic stress disorder: meta-analysis on the incidence and relative risk of deterioration, adverse events and serious adverse events. Br J Psychiatry. (2022) 221:658–67. doi: 10.1192/bjp.2022.111, PMID: 35959698

[24] Stingl M Hanewald B Kruse J Sack M . Positive side effects in trauma-focusing PTSD treatment: Reduction of attendant symptoms and enhancement of affective and structural regulation. psychol trauma: theory research Pract Policy. (2021) 13:713–21. doi: 10.1037/tra0000700, PMID: 32816515

[25] Hong J Park J-H . Efficacy of neuro-feedback training for PTSD symptoms: A systematic review and meta-analysis. Int J Environ Res Public Health. (2022) 19:13096–108. doi: 10.3390/ijerph192013096, PMID: 36293673 PMC9603735

[26] Mitchell JM Bogenschutz M Lilienstein A Harrison C Kleiman S Parker-Guilbert K . MDMA-assisted therapy for severe PTSD: A randomized, double-blind, placebo-controlled phase 3 study. Focus (American Psychiatr Publishing). (2023) 21:315–28. doi: 10.1176/appi.focus.23021011, PMID: 37404971 PMC10316215

[27] Reyes AT Bhatta TR Muthukumar V Gangozo WJ . Testing the acceptability and initial efficacy of a smartphone-app mindfulness intervention for college student veterans with PTSD. Arch Psychiatr Nurs. (2020) 34:58–66. doi: 10.1016/j.apnu.2020.02.004, PMID: 32248935

[28] Merz J Schwarzer G Gerger H . Comparative efficacy and acceptability of pharmacological, psychotherapeutic, and combination treatments in adults with posttraumatic stress disorder: A network meta-analysis. JAMA Psychiatry. (2019) 76:904–13. doi: 10.1001/jamapsychiatry.2019.0951, PMID: 31188399 PMC6563588

[29] Palmisano AN Meshberg-Cohen S Petrakis IL Sofuoglu M . A systematic review evaluating PTSD treatment effects on intermediate phenotypes of PTSD. psychol trauma: theory research Pract Policy. (2024) 16:768–83. doi: 10.1037/tra0001410, PMID: 36595460

[30] Cusack SE Coleman JA Rappaport LM Sheerin C . Moderation of improvement in self-efficacy following group psychotherapy for PTSD. Psychol Serv. (2019) 16:657–63. doi: 10.1037/ser0000260, PMID: 29963876 PMC6314904

[31] Vancappel A Hingray C Reveillere C El-Hage W . Disentangling the link between mindfulness and dissociation in PTSD: the mediating role of attention and emotional acceptance. J Trauma dissociation. (2024) 25:30–44. doi: 10.1080/15299732.2023.2231907, PMID: 37401352

[32] Wen X An Y Zhou Y Du J Xu W . Mindfulness, posttraumatic stress symptoms, and posttraumatic growth in aid workers: the role of self-acceptance and rumination. J Nerv Ment Dis. (2021) 209:159–65. doi: 10.1097/NMD.0000000000001275, PMID: 33273395

[33] Spidel A Daigneault I Kealy D Lecomte T . Acceptance and commitment therapy for psychosis and trauma: investigating links between trauma severity, attachment and outcome. Behav Cogn Psychother. (2019) 47:230–43. doi: 10.1017/s1352465818000413, PMID: 30012233

[34] Zhao Y An Y Sun X Liu J . Self-acceptance, post-traumatic stress disorder, post-traumatic growth, and the role of social support in Chinese rescue workers. J Loss Trauma. (2020) 25:264–77. doi: 10.1080/15325024.2019.1672935

[35] Roche L . An acceptance and commitment therapy-based intervention for PTSD following traumatic brain injury: a case study. Brain Inj. (2020) 34:290–7. doi: 10.1080/02699052.2019.1683896, PMID: 31657244

[36] Kelly MM Reilly ED Ameral V Richter S Fukuda S . A randomized pilot study of acceptance and commitment therapy to improve social support for veterans with PTSD. J Clin Med. (2022) 11:3482–97. doi: 10.3390/jcm11123482, PMID: 35743552 PMC9224981

